# Beliefs about optimal age and screening frequency predict breast screening adherence in a prospective study of female relatives from the Ontario Site of the Breast Cancer Family Registry

**DOI:** 10.1186/1471-2458-12-518

**Published:** 2012-07-12

**Authors:** Paul Ritvo, Sarah A Edwards, Gord Glendon, Lucia Mirea, Julia A Knight, Irene L Andrulis, Anna M Chiarelli

**Affiliations:** 1 Research, Prevention and Cancer Control, Cancer Care Ontario, 620 University Ave, Toronto, ON, M5G 2L7, Canada; 2 School of Kinesiology and Health Science, York University, 4700 Keele St, 138 Chemistry, Toronto, ON, M3J 1P3, Canada; 3 Dalla Lana School of Public Health, University of Toronto, 155 College St, Toronto, ON, M5T 3M7, Canada; 4 Ontario Cancer Genetics Network, Cancer Care Ontario, 620 University Ave, Toronto, ON, M5G 2L7, Canada; 5 Samuel Lunenfeld Research Institute, Mount Sinai Hospital, 600 University Ave, Toronto, ON, M5G 1X5, Canada; 6 Department of Molecular Genetics, University of Toronto, 1 King’s College Circle, Toronto, ON, M5S 1A8, Canada

**Keywords:** Breast cancer, Breast screening, Family history, Beliefs, Adherence

## Abstract

**Background:**

Although few studies have linked cognitive variables with adherence to mammography screening in women with family histories of breast and/or ovarian cancer, research studies suggest cognitive phenomena can be powerful adherence predictors.

**Methods:**

This prospective study included 858 women aged 30 to 71 years from the Ontario site of the Breast Cancer Family Registry with at least one first-degree relative diagnosed with breast and/or ovarian cancer. Data on beliefs about breast cancer screening and use of mammography were obtained from annual telephone interviews spanning three consecutive years. Self-reported mammogram dates were confirmed with medical imaging reports. Associations between beliefs about breast cancer screening and adherence with annual mammography were estimated using polytomous logistic regression models corrected for familial correlation. Models compared adherers (N = 329) with late-screeners (N = 382) and never-screeners (N = 147).

**Results:**

Women who believed mammography screening should occur annually were more likely to adhere to annual screening recommendations than women who believed it should happen less often (OR: 5.02; 95% CI: 2.97-8.49 for adherers versus late-screeners; OR: 6.82; 95% CI: 3.29-14.16 for adherers versus never-screeners). Women who believed mammography screening should start at or before age 50 (rather than after) (OR: 9.72; 95% CI: 3.26-29.02) were significantly more likely to adhere when compared with never-screeners.

**Conclusions:**

Study results suggest that women with a family history of breast cancer should be strongly communicated recommendations about initial age of screening and screening intervals as related beliefs significantly predict adequate adherence.

## Background

Given higher cancer detection rates [1] and increased odds of diagnosis with smaller, node-negative tumors with annual screening [2], earlier breast screening initiation (between 30 and 40 years) at shorter intervals (12 to 18 months) is recommended for women with family histories of breast cancers [3,4]. Because of these apparent screening advantages, it can be argued that screening adherence is especially important for this population.

Although social cognitive studies reveal beliefs to be strong adherence predictors across a spectrum of treatments and screening procedures [5-10], very few studies examined beliefs and screening adherence in women with familial breast cancer histories. While women with familial breast cancer histories tend to have better screening adherence [11-14], no data exists about how breast cancer screening beliefs can additionally influence adherence. Instead of beliefs, relevant knowledge has been emphasized as an adherence predictor, and while overlaps exist (between knowledge and beliefs), the knowledge construct operationalized in breast cancer screening studies does not often include identifications of specific beliefs. This is unfortunate as knowledge (alone) has yielded discouraging results in terms of both information acquisition and retrieval. In a large, multi-center study of 35 to 50 year old women, nearly all subjects had insufficient knowledge of screening effectiveness, although a majority responded correctly to questions about the purpose/consequences of mammography [15] and African American women (with and without family histories) had meager knowledge of several important risk factors (e.g. age, early menarche, late menopause) although correctly cited family history as one risk factor [16]. Since an adequate ‘fund’ of breast cancer-related knowledge appears an elusive goal in efforts to increase screening rates, focus might be better placed on promoting retention of a more limited set of specific beliefs that prove most predictive of screening adherence. For guidance as to which beliefs might be emphasized, we reviewed findings indicating that women who could not recall recommended screening intervals were significantly less likely to adhere [11,13,17,18]. As such our guiding hypothesis was that the beliefs most predictive of screening adherence would revolve around screening intervals and age of initiation. Those two beliefs answer fundamental questions about *when* to begin screening and *how long* to wait between screening events.

## Methods

### Study population

A cohort of female relatives of incident cases of invasive breast cancer was selected from the Ontario site of the Breast Cancer Family Registry (BCFR) funded by the United States National Cancer Institute. A description of the BCFR and the Ontario site of the BCFR have been published [19]. In brief, pathologically confirmed cases of invasive breast cancer (probands) diagnosed between 1996 and 1998 were identified from the population-based Ontario Cancer Registry (OCR). Physicians were contacted to obtain permission to contact their patients who were mailed a cancer *Family History Questionnaire.* Respondents meeting a defined set of family history criteria and a random sample (25%) of those not meeting the criteria were asked to participate in the Ontario site of the BCFR. Of those identified at this stage as eligible, 72% (1851/2587) participated.

Probands identified from the OCR were asked for address information and permission to contact specific living relatives (those affected with breast, ovarian, or certain other cancers, and their first degree relatives). An invitation letter to participate in the Ontario site of the BCFR was sent to relatives. Those agreeing to participate were sent an *Epidemiology Questionnaire* between 1998 and 2004. Our study was conducted a few years after the initial recruitment of relatives. For our study, we identified all female relatives who had: 1) enrolled in the Ontario site of the BCFR; 2) completed an *Epidemiology Questionnaire;* 3) been unaffected by breast cancer at the time of the proband’s diagnosis date; and 4) were alive and between the ages of 30 and 69 years as of January 1^st^, 2006. Sample size for our study was limited to the number of female relatives participating in the BCFR study, so power calculations were performed assuming we would have an 80% response rate from eligible women. From the 3374 participating female relatives, 2066 (61%) were residents of Ontario and 1471 (71.2%) of these women met our study inclusion criteria. Women participating provided written informed consent and this study was approved by the Research Ethics Boards of Mount Sinai Hospital and the University Health Network.

### Data collection

Information was obtained from three questionnaires. Eligible women were sent a baseline *Personal History and Screening Questionnaire* between November 2005 and March 2007. Those who were contacted and agreed to be interviewed were followed up annually for two years using the *Year 1 and Year 2 Follow-up Personal History and Screening Questionnaire*. An introductory letter and copy of each questionnaire were sent approximately two weeks before being contacted by phone. This allowed time for participants to recall specific dates and events and allowed referral to the questionnaire during the interview.

The baseline questionnaire collected key demographic characteristics, detailed information on breast cancer screening examinations and women’s beliefs about breast cancer screening. At each follow-up interview, women were asked whether they had a mammogram since their last contact. Women who reported having a mammogram were asked to give either the dates (month and year) of, or their age at, their last mammogram and the clinic or hospital where it was performed. In addition, for women who provided written consent to access their imaging report, the date and reason for the mammogram were abstracted.

### Definitions of breast cancer screening beliefs

Data on beliefs about breast cancer screening were obtained from questions concerning mammographic frequency (every 6 months; yearly; every two years; every three years) and initial age for mammographic screening (less than 30, 30-39; 40-49; 50-59 years). Women were also asked about the importance (extremely; very; somewhat; not at all) of several risk factors: high fat diet, alcohol use, lack of exercise, and family history. Finally, women rated the likeliness (very likely; somewhat; not very; not at all) of screening tests (mammogram and physical breast exam) finding breast cancer.

### Definition of annual screening adherence

Information on time-since-last-mammogram and the reason for mammographic examination obtained from the imaging report were combined to determine adherence with an annual screening recommendation. A mammogram was considered a screening mammogram if the indication on the imaging report was given as routine or regular screening and non-screening if the mammogram was performed because of a reported symptom or breast problem. Adherence was defined as returning within 18 months of a woman’s first reported screening mammogram which may have been reported in their baseline or year 1 follow-up interview.

### Definition of family history risk

Family history risk of breast and/or ovarian cancer was based on information collected from the *Family History Questionnaire* completed by the relative’s proband using a modified definition of previously referenced groups for familial breast cancer risk [3,20]. Women were considered to have a low familial risk if they had only one first-degree relative diagnosed with breast cancer after the age of 40. Women were considered to have a moderate familial risk if they had: 1) a self-reported Ashkenazi Jewish background; and/or 2) one first-degree relative with breast cancer diagnosed before the age of 40; or 3) one first-degree relative with ovarian cancer; or 4) one first-degree relative with breast cancer diagnosed after the age of 40 and two or more second-degree relatives with breast cancer diagnosed at any age. Finally, women were considered to have a high familial risk if they had: 1) two or more first-degree relatives with breast and/or ovarian cancer diagnosed at any age; and/or 2) one or more first-degree relative(s) with both breast and ovarian cancer diagnosed at any age; and/or 3) one or more first-degree relative(s) diagnosed with bilateral breast cancer at any age; and/or 4) a personal history of ovarian cancer.

### Definition of demographic and health characteristics

Demographic questions included date of birth from which age at interview was calculated (under 50 years; 50 years or older), highest level of education achieved (high school or less; some college/university/vocational/technical; bachelor’s degree or higher) and current marital status (married or common law; single, widowed, divorced, or separated). Information on health practices included a question on the number of yearly visits to health professionals on average in the last two years (once a year or less; 2 to 3 times per year; 4 or more times per year).

### Statistical analyses

Polytomous logistic regression analyses were used to examine associations between demographic characteristics, health practices, familial risk, breast cancer screening beliefs and adherence with annual screening within 18 months of the first reported mammogram, adjusted for age at baseline interview. Breast cancer screening belief questions were also adjusted by education, visits to health professionals and family history risk. Adherers were compared to late-screeners and never-screeners in each model. Models including interactions between each belief question and age or familial risk were examined to assess whether they were potential effect modifiers for breast cancer screening and beliefs. As no interaction was evident, no stratified analyses were performed. Since many study participants were members of the same family and could have similar cancer screening behaviors, a robust variance estimate was used to adjust for potential correlation due to family clustering [21,22]. Adjusted odds ratios (OR) and 95% confidence intervals (CI) were calculated. All ‘don’t know’ responses and refusals were excluded from the analyses. Analyses were conducted using SAS [23] and all reported P values are for two-sided alternatives with values <0.05 considered significant.

## Results

Of the 1471 eligible women sent a baseline *Personal History and Screening Questionnaire*, 1309 (89.0%) were contacted, 1071 (81.8%) agreed to participate and 69 women were ineligible (n = 37 had a breast cancer diagnosis and n = 32 did not have a first-degree relative with breast and/or ovarian cancer). Of the 1002 women sent a *Year 1 Follow-up Personal History and Screening Questionnaire*, 942 (94.0%) were interviewed. Of the 936 women without breast cancer sent a *Year 2 Follow-up Personal History and Screening Questionnaire*, 856 (91.5%) were interviewed. Women who reported having a mammogram (817 at baseline, 558 at year 1 and 540 at year 2) were asked to provide written consent for access to their medical imaging report. In total, 776 (95.0%), 536 (96.0%) and 511 (94.6%) consented to share their mammogram report after completing the baseline, year 1 and year 2 questionnaires, respectively; and of these, 774 (99.7%), 453 (84.6%) and 497 (97.3%) reports were received.

**Figure 1:**
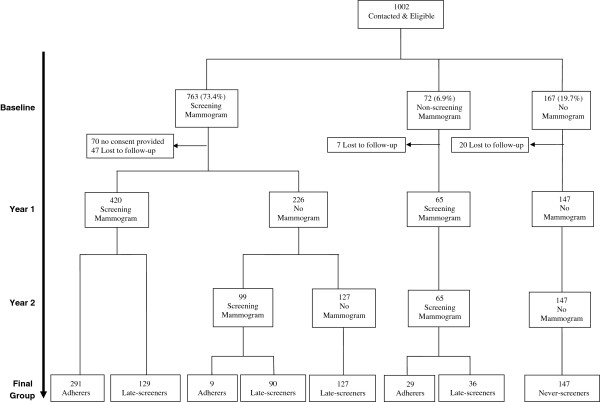
Tree diagram of women included in the study.

Of the 646 eligible women with a baseline screening mammogram (Figure 1), 420 reported a screening mammogram at year 1 (291 within 18 months and 129 beyond 18 months). Of the remaining 226 women with no screening mammogram at year one, 99 reported a screening mammogram at their second year interview (9 within 18 months and 90 beyond 18 months) and 127 did not report one. There were 65 women reporting no baseline screening mammogram but screening mammograms at year 1 and 2, 29 of which returned within 18 months and 36 beyond 18 months. Overall, 711 women reported at least two screening mammograms, of which 329 (46.3%) were adherers and 382 (53.7%) were late-screeners. There were 147 women who reported having no mammogram at all three interviews (baseline, year 1 and year 2) and these women were considered never-screeners.

The final sample size included 858 women from 569 unique families of which 381 (67.0%) had one family member, 121 (21.3%) had two family members and 67 (11.7%) had three to seven family members. The majority of adherers and late-screeners were aged 50 years or older (74.1% and 57.1%, respectively) whereas the majority of never-screeners were under age 50 (87.8%) (Table 1). For adherers, a similar distribution was found of women in the low, moderate and high familial risk groups, whereas, nearly 50% of the late-screeners and more than 70% of the never-screeners were at low familial risk. Moderate (OR: 1.55; 95% CI: 1.06-2.27) or high (OR: 1.48; 95% CI: 1.04-2.12) versus low familial risk was significantly associated with a woman returning for screening within 18 months versus beyond 18 months. Similarly, moderate (OR: 2.47; 95% CI: 1.38-4.41) and high (OR: 2.01; 95% CI: 1. 06-3.80) versus low familial risk were significantly associated with women returning within 18 months versus never being screened. A majority of the women had more than a high school education, were married and had visited a health professional three times or less per year in all three screening groups.

**Table 1:** Adjusted odds ratios (ORs) with 95% confidence intervals (CI) for adherence to annual breast screening by demographic, family history risk and visits to health professionals for female relatives from the Ontario site of the Breast Cancer Family Registry

**Demographic, Health and Screening Characteristics**	**Screening mammogram**			**Adherers vs. Late-screeners**	**Adherers vs. Never-screeners**
	**Adherers (N = 329)**	**Late-screeners (N = 382)**	**Never-screeners (N = 147)**	Adjusted^*^OR 95% CI	Adjusted^*^OR 95% CI
Age at baseline interview (years)					
30-39	11 (3.4)	30 (7.8)	97 (66.0)		
40-49	74 (22.5)	134 (35.1)	32 (21.8)		
50-59	133 (40.4)	136 (35.6)	12 (8.1)		
≥ 60	111 (33.7)	82 (21.5)	6 (4.1)		
Family History Risk					
Low	124 (37.7)	189 (49.5)	103 (70.1)	1.00	1.00
Moderate	92 (28.0)	89 (23.3)	24 (16.3)	1.55 (1.06, 2.27)^†^	2.47 (1.38, 4.41)^‡^
High	113 (34.3)	104 (27.2)	20 (13.6)	1.48 (1.04, 2.12)^δ^	2.01 (1.06, 3.80)^δ^
Education level					
High school or less	122 (37.1)	115 (30.2)	22 (14.9)	1.00	1.00
Some college/university/ vocational/technical school	124 (37.7)	166 (43.6)	57 (38.8)	0.86 (0.60, 1.24)	1.38 (0.69, 2.76)
Bachelor’s degree or higher	83 (25.2)	100 (26.2)	68 (46.3)	1.02 (0.68, 1.54)	1.08 (0.55, 2.12)
Married or Common-Law					
Yes	272 (82.7)	314 (82.2)	126 (85.7)	1.00	1.00
No	57 (17.3)	68 (17.8)	21 (14.3)	0.90 (0.60, 1.35)	1.32 (0.66, 2.66)
Visits to Health Professionals					
Once a year or less	109 (33.1)	134 (35.1)	55 (37.4)	1.00	1.00
2-3 times per year	148 (45.0)	142 (37.2)	61 (41.5)	1.25 (0.88, 1.77)	1.23 (0.73, 2.08)
4 or more times per year	72 (21.9)	106 (27.7)	31 (21.1)	0.76 (0.51, 1.14)	1.21 (0.67, 2.20)

^*^Adjusted by age at interview.

^†^*p*-value = 0.024.

^‡^*p*-value = 0.002.

^δ^*p*-value = 0.032.

Women who indicated a belief in annual mammogram screening were significantly more likely to be screening adherent (OR: 5.02; 95% CI: 2.97-8.49 for adherers versus late-screeners and OR: 6.82; 95% CI: 3.29-14.16 for adherers versus never-screeners) than women who believed in a lesser frequency (Table 2). When adherers were compared with never-screeners, women who believed mammography should start for higher risk women before age 50 were significantly more likely to adhere than those who thought it should start at ≥ 50 years (OR: 9.72; 95% CI: 3.26-29.02). Screening adherence was not associated with beliefs about breast cancer risk factors or beliefs about the likeliness of a mammogram finding breast cancer when adherers were compared with late-screeners or never-screeners.

**Table 2:** Adjusted odds ratios (ORs) with 95% confidence intervals (CI) for adherence to annual breast screening by breast screening belief factors for female relatives from the Ontario site of the Breast Cancer Family Registry

**Breast Cancer Beliefs Factors**	**Screening mammogram**			**Adherers vs. Late-screeners**	**Adherers vs. Never-screeners**
	**Adherers (N = 329)**	**Late-screeners (N = 382)**	**Never-screeners (N = 147)**	Adjusted^*^OR 95% CI	Adjusted^*^OR 95% CI
Frequency screening mammogram					
Less often (once every two years or more)	23 (7.0)	97 (26.6)	39 (30.0)	1.00	1.00
At least once per year	306 (93.0)	267 (73.4)	91 (70.0)	5.02 (2.97, 8.49)^†^	6.82 (3.29, 14.16)^†^
Age start screening mammogram					
≥50	26 (8.1)	28 (7.6)	11 (7.7)	1.00	1.00
<50	296 (91.9)	341 (92.4)	132 (92.3)	1.38 (0.75, 2.55)	9.72 (3.26, 29.02)^†^
Importance of Breast Cancer Risk Factors					
High fat diet					
Not at all/Somewhat important	101 (37.5)	144 (44.7)	58 (44.3)	1.00	1.00
Very/Extremely important	168 (62.5)	178 (55.3)	73 (55.7)	1.22 (0.87, 1.70)	1.23 (0.74, 2.06)
Alcohol use					
Not at all/Somewhat important	172 (63.9)	221 (68.6)	93 (71.0)	1.00	1.00
Very/Extremely important	97 (36.1)	101 (31.4)	38 (29.0)	1.17 (0.82, 1.67)	1.20 (0.69, 2.07)
Lack of exercise					
Not at all/Somewhat important	105 (39.1)	147 (45.7)	58 (44.3)	1.00	1.00
Very/Extremely important	164 (60.9)	175 (54.3)	73 (55.7)	1.21 (0.87, 1.68)	1.50 (0.89, 2.52)
Family history of breast cancer					
Not at all/Somewhat important	22 (8.2)	22 (6.8)	10 (7.6)	1.00	1.00
Very/Extremely important	247 (91.8)	300 (93.2)	121 (92.4)	0.90 (0.47, 1.71)	1.32 (0.45, 3.87)
Likeliness mammogram finding breast cancer					
Not at all/not very likely	122 (37.1)	150 (39.8)	55 (37.9)	1.00	1.00
Very/somewhat likely	207 (62.9)	227 (60.2)	90 (62.1)	1.10 (0.79, 1.53)	1.24 (0.75, 2.05)

^*^Adjusted by age at interview, education, visits to health professionals, family risk and were corrected for familial clustering.

^**†**^*p*-value = <0.0001.

## Discussion

This study found specific beliefs about breast screening associated with screening adherence. Women who believed in annual screening were nearly five times more likely to adhere to screening (within 18 months) than women who believed they should return less often. Subjects who believed they should start screening before 50 years of age (rather than afterwards) were nearly 10 times more likely to adhere than those who were never-screened.

Studies of average risk women between 50-69 years participating in screening programs and two broader population surveys including women 40-75 years have shown that a high proportion believe in the appropriate frequency of screening. Between 77.2% and 94.1% reported beliefs that women should be screened every 1-2 years [11,13,18,24]. These data become more consequential with findings that beliefs about screening guidelines predict screening adherence in women with a family history of breast cancer. Our findings are consistent with studies that examined this relationship in average risk women over age 50 where women who reported screening should occur every 1-2 years (in the US and Australia where biennial mammography screening is recommended) were significantly more likely to adhere than women who reported mammograms should occur less often [17,18].

The same association between beliefs regarding screening timeliness and adherence was supported in a previous study of average-risk women, 50-75 years, attending the Ontario Breast Screening Program. We found those who believed in less frequent than biennial screening were significantly less likely to biennially screen [24]. These findings underscore the importance of communicating a screening interval recommendation to women at increased risk for breast cancer with sufficient strength that they develop strong beliefs about the need to screen at the specific interval recommended.

We found no significant association between women believing a mammogram was likely to find breast cancer and screening adherence. This contrasts with previous research that found women who believe mammograms are effective are more likely to adhere [11,14]. One reason for the inconsistency may be that our subjects were high risk women compared to the other studies that included average risk women.

Previous research in average risk women has shown that referring physicians have an important influence on women, in terms of both initially attending screening and subsequent screening adherence [12,13,25-28]. A recent Australian study of women with a strong family history of breast cancer confirms this as women who attended mammography screening less often than recommended in national guidelines were less likely to have received a screening recommendation compared to appropriate screeners [29]. Physicians and other health professionals should be encouraged to not only discuss screening benefits but to strongly emphasize screening timeliness, particularly to those at high risk, because our findings suggest cognitions relating to screening intervals may be key in actual (timely) screening adherence.

To our knowledge, this is the first study to examine the impact of beliefs about breast cancer screening on screening adherence in women at high familial risk. Because our study includes a large cohort of female relatives of population-based breast cancer cases, there was sufficient power to examine associations and minimize self-referral bias. An additionally important strength is our prospective examination of the impact of beliefs on adherence. Finally, we obtained actual mammography dates to calculate adherence rather than relying on self-reported data. While self-reported mammography data has been found to be accurate for determining whether a woman has had a mammogram, it is less accurate in determining the interval since last mammogram [30].

Our study limitations include being unable to determine adherence or non-adherence in approximately 7.0% of women who did not provide consent to access their mammogram report and in 7.4% of women who were lost to follow-up. In addition, self-reported data was used for determining family history of breast cancer. Previous research has demonstrated that patient-reported family cancer histories for first-degree relatives are accurate for breast cancer risk assessments, although accuracy is somewhat lower for second-degree relatives [31]. Finally, our study findings may have limited generalizability as participants were family members of breast cancer cases identified from a population-based registry in Ontario, Canada where universal health care coverage (covering all physician referred screening at any age) and an organized breast cancer screening program for women 50 to 74 years of age are available.

## Conclusions

Altogether, this study found that believing in the appropriate frequency and age range to begin breast screening was associated with significantly increased adherence to annual screening for women at familial risk. In contrast, beliefs about the importance of risk factors for breast cancer were not associated with adherence. Study results suggest women with a family history of breast cancer should continue to be informed about breast cancer screening benefits, but special emphasis should be placed on strongly communicating the recommended frequency and age to begin screening for women at increased familial risk. If women develop strong beliefs about optimal frequency and age to begin screening, our study indicates their breast screening adherence will be significantly improved.

## Abbreviations

BCFR, Breast Cancer Family Registry; OCR, Ontario Cancer Registry; OR, Odds Ratio; CI, Confidence Interval.

## Competing interests

The authors declare that they have no competing interests.

## Authors’ contributions

PR, AMC, GG, JAK and ILA conceived of the study and participated in its design and coordination. SAE, AMC, PR and LM performed statistical analyses and interpretation of the data. PR, SAE and AMC drafted the manuscript and GG, LM, JAK and ILA helped revise the manuscript. All authors read and approved the final manuscript.

## Grant support

This research was supported by the Canadian Breast Cancer Research Alliance (Grant 016270). This work was also supported by the National Cancer Institute, National Institutes of Health under RFA-CA-06-503 and through cooperative agreements with members of the Breast Cancer Family Registry and Principal Investigators, including Cancer Care Ontario (U01 CA69467). The content of this manuscript does not necessarily reflect the views or policies of the National Cancer Institute or any of the collaborating centers in the Breast CFR, nor does mention of trade names, commercial products, or organizations imply endorsement by the US Government or the Breast CFR.

## Pre-publication history

The pre-publication history for this paper can be accessed here:

http://www.biomedcentral.com/1471-2458/12/518/prepub
